# Social Interaction Is Less Rewarding in Adult Female than in Male Mice

**DOI:** 10.3390/brainsci13101445

**Published:** 2023-10-10

**Authors:** Anna E. Granza, Inês M. Amaral, Diogo G. Monteiro, Ahmad Salti, Alex Hofer, Rana El Rawas

**Affiliations:** 1 Division of Psychiatry I, Department of Psychiatry, Psychotherapy, Psychosomatics and Medical Psychology, Medical University Innsbruck, 6020 Innsbruck, Austria; 2 University Clinic of Ophthalmology and Optometry, Kepler University Hospital, Johannes Kepler University Linz, Krankenhausstrasse 5, 4020 Linz, Austria

**Keywords:** social interaction, reward, neuropeptide, αCaMKII, sex difference, mice

## Abstract

(1) Background: Positive social relationships are essential for mental and physical health. However, not all individuals experience social interaction as a rewarding activity. (2) Methods: Social interaction reward in mice can be assessed by social conditioned place preference (CPP). The aim of this study is to investigate sex-dependent differences in the neurological underpinnings underlying social versus non-social phenotypes, using adult male and female C57BL/6J mice. (3) Results: Adult female mice expressed significantly less social reward than males from the same strain. Accordingly, pairs of male mice spent more time interacting as compared to female pairs. Subsequently, we analyzed neuropeptides previously reported to be important regulators of social behavior such as oxytocin, vasopressin, and orexin, in addition to Ca^2+^/calmodulin-dependent protein kinase II (αCaMKII), shown to be involved in social reward. Levels of neuropeptides and αCaMKII were comparable between males and females in all investigated regions. Yet, a significant negative correlation was found between endogenous oxytocin expression and social reward in female pairs. (4) Conclusions: Sex differences in the prevalence of many mental health disorders might at least in part be due to sex differences in social reward. Therefore, more research is needed to unravel the candidate(s) underlying this behavioral difference.

## 1. Introduction

Impaired social interaction is a hallmark symptom of many mental health disorders (MHD). In fact, facing difficulties in social relations or being socially isolated is known to increase the risk of developing MHD and strongly impacts symptom progression and health outcomes [1].

In humans and other social animals such as monkeys and rodents, positive social interaction and social support enhance resilience to stress and drugs of abuse-associated effects [2,3,4] and help protect mental health [5]. However, some individuals display a diminished interest in social relationships [6] and do not experience social interaction as a rewarding activity.

In laboratory rodents, the rewarding effects of social interaction can be assessed using a standard preclinical behavioral model—Conditioned Place Preference (CPP), established via classical conditioning [7]. Social reward CPP is obtained when animals show a preference towards a social interaction-associated context after several conditioning sessions comprised of social interaction between two sex- and weight-matched animals in a specific context of a CPP apparatus [8,9,10,11,12,13,14,15]. Interestingly, the rewarding value of social interaction seems to be slightly different when comparing rats and mice. In a study performed by Kummer et al., 85% of male rats tested developed social interaction CPP [16]. When comparing different C57BL6 mouse substrains, Pinheiro et al. observed that only 48% of the NIH sub-strain mice developed a preference to social interaction, in contrast to 62% of the Jackson sub-strain mice [17]. Furthermore, social interaction as a composite stimulus has a wide range of distinct sensory modalities, from which physical contact (touch) appears to be the most rewarding [18]. Species- and strain-dependent differences have also been observed concerning this particular component of social interaction. Rats spent, on average, more than 79% of social interaction conditioning sessions in direct contact and fully engaged in friendly (“agonistic”) social behavior as no episodes of hostile behavior, i.e., boxing or biting, were observed [16]. In contrast, mice only spent an average of 17% of time in direct contact, also with no signs of aggression [16]. Even though rats spent significantly more time in direct contact with the social interaction partner than mice, social interaction was mostly rewarding for both species [16]. Given these findings, the questions that arise are: why social interaction is not equally rewarding for all mice and whether we can identify the neurological bases of these social versus non-social phenotypes.

Research on the neurobiology of social interaction reward using laboratory rodents has mostly studied males, with only a few comparative studies [10,19,20,21], thus contributing to the knowledge gap concerning sex-dependent differences in this type of reward. In fact, despite behavioral similarities between males and females, the mechanism(s) used to respond to social and emotional challenges and opportunities seem to be different [22].

In this study, we report evidence in an animal model that social interactions with same-sex conspecifics are significantly less rewarding in adult females than in adult males. These studies were performed in adult male and female C57BL/6J mice: the most widely used inbred strain. Hence, we aimed to investigate sex-dependent differences in the neurological underpinnings underlying social versus non-social phenotypes. For that reason, we explored neuropeptides previously reported to be important regulators of social behavior such as oxytocin and vasopressin [23], which are mainly synthetized in the hypothalamus, and orexin [24,25], produced in the lateral hypothalamic area (LH). These neuropeptides have not, to our knowledge, previously been investigated in males and females C57BL6J mice using a social interaction CPP protocol. In addition, based on our previous findings concerning the involvement of Ca^2+^/calmodulin-dependent protein kinase II (CaMKII) in social interaction reward [26], we evaluated αCaMKII expression levels in the nucleus accumbens (NAc) and the ventral tegmental area (VTA), two central brain regions of the reward circuitry. Understanding sex differences in the mechanisms of social reward is crucial as impairments in social reward are linked with a variety of psychiatric disorders, many of which are sex-dependent in terms of prevalence and predisposition.

## 2. Materials and Methods

### 2.1. Animals

Adult (3–5 months old) male (*n* = 18) and female (*n* = 18) C57BL/6J mice were singly housed upon arrival to the animal facility and remained isolated throughout the entire experiment with ad libitum access to water and food. All behavioral experiments were conducted during the light period of a 12 h-light/dark cycle. Experiments were approved by the Austrian National Animal Experiment Ethics Committee (BMBWF-66.011/0138-V/3b/2019 and 2021-0.435.536).

### 2.2. Conditioned Place Preference (CPP)

#### 2.2.1. Apparatus

CPP was conducted in a three-chamber apparatus (64 cm width × 32 cm depth × 31 cm height) made of unplasticized polyvinyl chloride. The middle (neutral) compartment of the CPP apparatus (10 × 30 × 30 cm) had white walls and floor. Two removable doors connected the middle compartment to two conditioning compartments (25 × 30 × 30 cm) with walls that had either vertical or horizontal black and white stripes and stainless-steel floors with either 56 slits (4.2 × 0.2 cm) or 168 holes (diameter 0.5 cm). Between each session, the apparatus was cleaned with a 70% ethanol solution. All experiments were performed under a neon ceiling light (58 W, 1 m distance), as previously described [16,17,27]. All sessions were recorded with a video camera placed above the apparatus. Behavioral analysis was performed using the ANY–maze Video Tracking Software (version 7.1) (Stoelting Europe, Dublin, Ireland).

#### 2.2.2. Social Interaction CPP

Acquisition: On Day 1, a pretest was conducted to evaluate the mice’s natural preference for a compartment of the CPP. For this, mice were placed in the middle compartment of the apparatus for 15 s (habituation), after which the doors were removed and the animals allowed to explore freely for 30 min. The time mice spent in each of the conditioning compartments was assessed. On Days 2–5, conditioning sessions were conducted by pairing the less preferred compartment (in the pretest) with social interaction conditioning. Briefly, 30-minute sessions were performed in which mice were either placed alone in a compartment (neutral stimulus) or placed in the opposite compartment with a previously assigned sex- and weight-matched mouse for social interaction (that remained the same for the entire experiment). The removable doors were closed and accordingly, mice only had access to the compartment in which they had been placed. For each stimulus, a total of 4 conditioning sessions were performed (1 per day; morning and afternoon sessions, separated by at least 4 h).

Test: On Day 6, the expression of preference for social interaction CPP was evaluated in a session similar to the pretest. Mice were again allowed to explore the entire apparatus for 30 min and the time spent in each compartment was recorded. A preference score (PS) was calculated by subtracting the time mice spent in the social interaction-paired compartment in the test minus the time spent in the same compartment in the pretest. A social phenotype was determined when the preference score of a mouse is higher than zero seconds (PS > 0), whereas a non-social phenotype was determined when the preference score of a mouse is lower than zero seconds (PS < 0).

#### 2.2.3. Analysis of Social Interaction Components

i. Time in social contact

Social interaction conditioning sessions were recorded with a video camera and the total time a pair of mice spent in direct physical contact in the last session was measured [16].

ii. Hierarchy

Following a scoring system [17,28], all mice used in this study were assigned a hierarchy score according to the signs of dominance or subordination displayed in the last social interaction conditioning session (conditioning session 4). Briefly, a hierarchy score of h3 (aggressive dominance) was assigned when a mouse performed 3 consecutive attacks to the paired mouse (characterized by aggressive grooming, biting, and chasing); h2 (passive dominance) was defined as consistent threatening displacement by one mouse, including upright or sideways postures; h0 (subordination) was assigned when there was clear retreat/fleeing posture displayed by a mouse; h1 (draw) was assigned to pairs that showed none of the signs listed above.

### 2.3. Quantitative Real-Time PCR

Animals were sacrificed 30 min after the test by an overdose IP injection (2 mL/kg) of sodium pentobarbital (300 mg/mL). Brains were removed and immediately frozen in −40 °C isopentane. NAc, LH, hypothalamus, and VTA were punched out of thaw-mounted coronal 100-μm sections at −15 °C in a cryostat (Leica CM3050 S) using a sample corer (Fine Science Tools, Foster City, CA, USA, 15 G).

Total RNA was isolated from the dissected brain regions using the Monarch^®^ Total RNA Miniprep Kit (#T2010G, New England BioLabs, Ipswich, MA, USA) according to the manufacturer’s protocol. RNA purity and concentration were evaluated using a NanoDrop spectrophotometer (peqlab Biotechnologies, Erlangen, Germany). In total, 200 ng of RNA was reverse transcribed into cDNA using the OneScript^®^ Plus cDNA Synthesis Kit from Applied Biological Materials Inc. Richmond, BC, Canada (abm) in a total volume of 20 μL.

After dilution with 80 μL of water, 5 μL of the diluted cDNA was used as a template for amplification (duplicates) using the SsoAdvanced Universal SYBR^®^ Green Supermix (Bio–Rad Laboratories, Hercules, CA, USA). Real-time polymerase chain reaction quantification (qRT-PCR) was performed on a CFX96 Touch Real-Time PCR Detection System (Bio–Rad Laboratories) using the following cycle settings: 30 s 95 °C, 40 cycles of 95 °C for 5 s, and 60 °C for 30 s. All PCR primers were designed using PrimerSelect 7.1.0 software (DNASTAR Lasergene). The efficiency of the primers was verified using a 2-fold serial dilution of cDNA and melt-curve analysis. The cycle threshold (Ct) and ΔCt values were calculated using CFX Maestro™ software (version 2.2) and GAPDH as a reference gene. Relative changes in gene expression (fold change) were determined through the 2^−ΔΔCt^ method. Primers used in this study are detailed in Table 1.

### 2.4. Statistical Analysis

Statistical analysis was performed using GraphPad Prism 9.4.1 software. Data are expressed as the mean ± SEM, and *p* values < 0.05 were considered statistically significant. The statistical significance of the behavioral data was tested using a two-tailed unpaired Student’s *t*-test, after passing the normal distribution tests. To test the statistical significance of the molecular data, a two–way ANOVA was performed. Linear correlations between 2 variables of the same experimental group were tested using the Pearson correlation coefficient (r).

## 3. Results

### 3.1. Social Interaction Is Less Rewarding in Adult Females Compared to Adult Males

**Figure 1:**
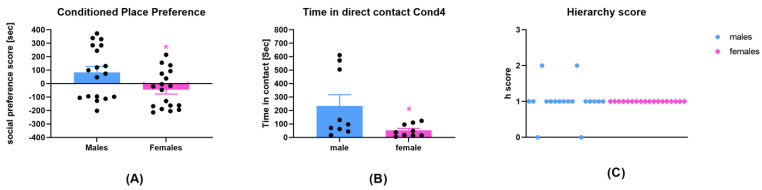
Behavioral analysis of the social profile of male and female mice. (**A**) Conditioned place preference represented as social preference score in seconds for males (*n* = 18) and females (*n* = 18). (**B**) Time in direct contact during Conditioning Session 4 of males (9 pairs) and females (9 pairs). (**C**) Hierarchy score during Conditioning Session 4 of males (9 pairs) and females (9 pairs). * unpaired student’s *t*-test; *p* < 0.05 male vs. female.

The social CPP of male and female adult mice was compared after four conditioning sessions with sex- and weight-matched conspecifics. Males expressed higher social preference scores than females did (unpaired *t*-test two-tailed male vs. female, *p* = 0.0283; t (34) = 2.290; *n* = 18 per group)—Figure 1A. Whereas 61% of male mice expressed social CPP, only 33% of female mice did. This difference was reflected in the time mice spent interacting together in the last social conditioning session (Conditioning Session 4). Indeed, male mice spent significantly more time in direct contact than female mice did (unpaired *t*-test two-tailed male vs. female, *p* = 0.0469; t (16) = 2.154; *n* = 9 pairs per group)—Figure 1B. The average PS obtained in male mice (83 s ± 45, *n* = 18) is comparable to the average PS of two batches of male rats conditioned with social interaction (80 s ± 14; 86 s ± 18) from our previous study [12]. In the same study, male rats not exposed to social conditioning displayed a PS of −10 s ± 22. However, the only difference is the higher variability in the PS of mice compared to rats. Indeed, whereas 100% of rats express social interaction reward from the study in [12], only 11 out of 18 male C57bL/6J mice, equivalent to 61%, show social CPP. These results are in line with previous studies showing that a high percentage of Sprague Dawley rats, i.e., 85%, and a relatively lower percentage of C57BL/6J, i.e., 62%, express social interaction CPP [16,17].

To check whether the non-social profile in females was due to differences in hierarchy in the social pairs, i.e., dominance/subordinate status, we evaluated the hierarchy scores in male and female pairs. All female mice were socially equal and were ranked h1 (Figure 1C). In male mice, only two pairs were ranked h0–h2, thereby suggesting a passive dominance–subordination status (Figure 1C).

### 3.2. Neuropeptides Expression in Males vs. Females

In order to investigate the mechanisms underlying the different social profiles of males and females, we compared the levels of oxytocin and vasopressin in the hypothalamus and of orexin in the LH of male and female mice showing a social vs. a non-social profile.

**Figure 2:**
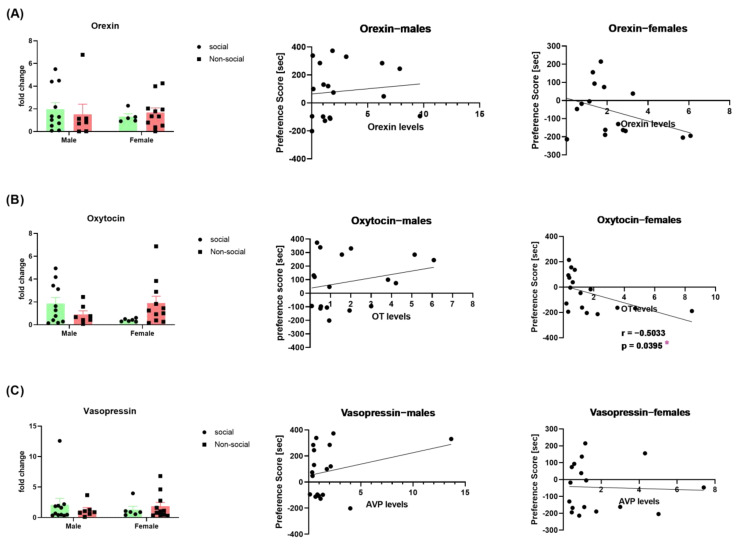
Neuropeptides expression in the hypothalamus (oxytocin and vasopressin) and the LH (orexin) of male and female mice. Orexin (**A**) (*n* = 5–11 per group); oxytocin (**B**) (*n* = 6–11 per group), and vasopressin (**C**) (*n* = 6–11 per group) levels in social and non-social male and female mice; correlation between social preference score and neuropeptide expression in male (middle) and female (right) mice. * *p* < 0.05 significant correlation, Pearson r calculation.

Orexin levels were comparable between males and females with a social or a non-social profile [two-way ANOVA; social phenotype effect (F (1, 30) = 0.006879; *p* = 0.9345); sex effect (F (1, 30) = 0.1496, *p* = 0.7016); interaction (sex x social phenotype) (F (1, 30) = 0.3968, *p* = 0.5335)]. In neither males (*p* = 0.6528; r = 0.1139) nor females (*p* = 0.1492; r = −0.3777) did the orexin levels correlate with the social preference score—Figure 2A.

Similarly, oxytocin levels were comparable between males and females with a social or a non-social profile [two-way ANOVA; social phenotype effect (F (1, 31) = 0.2552; *p* = 0.6170); sex effect (F (1, 31) = 0.1778, *p* = 0.6761)]. Yet, the interaction (sex x social phenotype) was significant (F (1, 31) = 4.975, *p* = 0.0331)]. Oxytocin levels were not correlated with the social preference score of male mice (*p* = 0.3222; r = 0.2474) but negatively correlated with the social preference score of female mice (*p* = 0.0395; r = −0.5033)—Figure 2B.

Vasopressin levels in the hypothalamus were comparable between males and females with a social or a non-social profile [two-way ANOVA; social phenotype effect (F (1, 31) = 0.006051; *p* = 0.9385); sex effect (F (1, 31) = 0.03849, *p* = 0.8457); interaction (sex x social phenotype) (F (1, 31) = 0.6820, *p* = 0.4152)]. Vasopressin levels were correlated with neither the social preference score of males (*p* = 0.2535; r = 0.2839) nor that of females (*p* = 0.8696; r = −0.04309)—Figure 2C.

### 3.3. αCaMKII Expression in Males vs. Females

**Figure 3:**
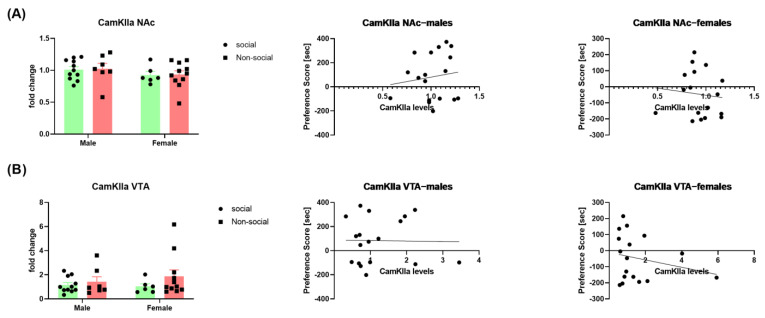
αCaMKII expression in the NAc and the VTA of male and female mice. αCaMKII levels in the NAc (**A**) (*n* = 6–11 per group) and the VTA (**B**) (*n* = 6–11 per group) in social and non-social male and female mice; correlation between social preference score and αCaMKII expression in male (middle) and female (right) mice.

CaMKII in the NAc has previously been shown to be involved in social interaction reward [26]. However, as both the NAc and the VTA are essential for reward processing, we evaluated the levels of αCaMKII in both regions. Similar to neuropeptide levels, the levels of αCaMKII were comparable between groups in either the NAc [two-way ANOVA; social phenotype effect (F (1, 31) = 0.02421; *p* = 0.8774); sex effect (F (1, 31) = 1.795, *p* = 0.1900); interaction (sex × social phenotype) (F (1, 31) = 0.0004688, *p* = 0.9829)] or the VTA [two-way ANOVA; social phenotype effect (F (1, 31) = 1.681; *p* = 0.2043); sex effect (F (1, 31) = 0.1544; *p* = 0.6970); interaction (sex × social phenotype) (F (1, 31) = 0.4952, *p* = 0.4869)]. Furthermore, no significant correlations were found between the social preference score and αCaMKII expression in both regions. This was true for male [NAc: (*p* = 0.5755; r = 0.1415); VTA: (*p* = 0.9482; r = −0.01651)] and female mice [NAc: (*p* = 0.6843; r = −0.1064); VTA: (*p* = 0.3771; r = −0.2288)]—Figure 3.

## 4. Discussion

Adult female C57BL6 mice expressed significantly less social reward than males from the same strain. This (in)ability to enjoy social interaction was reflected in the time of direct contact between pairs, i.e., pairs of male mice spent more time interacting as compared to female pairs. The high percentage of non-sociability in female mice was not due to the establishment of social dominance relationships [29], as all female pairs were socially equal.

It seems that female mice from the C56BL6 strain are less socially interactive than those from other mouse strains. Indeed, when comparing CD1 to C57BL6 female mice, the former have been shown to demonstrate significantly stronger social self-administration, as well as robust social seeking after social isolation [21]. Additionally, in the choice task between palatable food versus social interaction, CD1 mice preferred social interaction, whereas C57BL6 mice preferred food [21]. Likewise, CD1 but not C57BL6 mice demonstrated robust social CPP [21]. Our results add to these latter findings that male C57BL6 are significantly more potent in expressing social reward than their female counterparts. On the other hand, in a study conducted in Syrian hamsters, it was found that females find same-sex social interactions to be significantly more rewarding than males [19]. Further, these studies demonstrated that the activation of oxytocin receptors in the VTA plays a critical role in mediating the rewarding properties of social interactions in both males and females [19]. These data indicate that studies investigating sex differences in social reward may be essential for defining the basic mechanisms underlying the difference in the expression of social behavior.

Although prepro-orexin mRNA [30] and orexin-A protein [31] levels have been shown to be higher in adult female compared to male rats, we did not observe sex differences in the orexin expression levels after social interaction CPP. Our results are in agreement with [24], reporting that the total number of orexin immuno-reactive neurons was similar in male and female rats after exposure to social play. Interestingly, lower prepro-orexin mRNA levels were found in actively coping rats when compared with passively coping rats in a social defeat model [32]. Given that social interaction conditioning has anti-stress effects [12], a decrease in stress levels in female mice that expressed social interaction reward could lead to a decrease in orexin levels in females to the level of male mice, thereby masking any expected sex difference between males and females.

Oxytocin and vasopressin expression levels were found to be comparable between males and females. Accordingly, most studies did not find any sex differences in vasopressin mRNA expression in the hypothalamus of adult rats [33]. Moreover, depending on the species, oxytocin mRNA expression or immunoreactivity in the brain of several rodent species is similar in males and females [33]. Yet, in those rodent species that show a sex difference, oxytocin-immunoreactivity is consistently higher in females compared to males, suggesting a greater role of oxytocin in females [33]. In line with this suggestion, we found a negative correlation between endogenous oxytocin expression and social reward in female pairs.

It appears that vasopressin has a sex-specific role in other forms of social interaction than the one conducted in this study. An involvement of vasopressin has been described in partner preference formation [34] and social recognition [35], where vasopressin played a greater role in males than in females. Alternatively, vasopressin and oxytocin have been shown to regulate social play behavior in a sex-specific way at the juvenile age [36,37]. Coming back to the age factor, adult female rats show a lower social interest than males [38]. This is believed to be due to the fact that these female rats show lower forebrain oxytocin receptor binding densities than males [38]. Interestingly, quite in parallel with our findings, female social interest correlated negatively with oxytocin binding densities in the region of the central amygdala [38], suggesting thereby that the oxytocin system is differently involved in modulating the salience of social stimuli in males and females, possibly via different mechanisms [33].

We have previously shown that social interaction reward engages αCaMKII in the NAc [26] and expected that a differential αCaMKII expression might underlie the difference in expression of social reward in male and female mice. Yet, we found that αCaMKII expression was not different between males and females, neither in the VTA nor in the NAc. Most of the few studies about an involvement of CaMKII in social interaction focus on only one sex [26,39]. Yet, in a study using male and female mice with lower total forebrain αCaMKII levels, it was shown that both display aberrant behavioral phenotypes, including social interaction deficits [40], thereby decreasing the probability of a sex difference in the involvement of αCaMKII in social behaviors.

One limitation of this study is that we did not correlate the estrous cycle of female mice with the social or non-social profile. Interestingly, one study using female C57BL/6J mice has shown that there was a significant difference in three-chamber social interaction between female mice at different stages of their estrous cycle [41]. Females in the proestrus and estrus stages showed no preferential interest in a novel female age- and sex-matched mouse compared with an empty chamber, whereas female mice in the metestrus and diestrus stages performed similarly to males in this task, showing a greater interaction time with a novel age- and sex-matched mouse compared with an empty cage [41]. These findings suggest that, in our study, non-social female mice might be at the proestrus or estrus stage of their cycle whereas social female mice might be at the metestrus or diestrus stage of their cycle. Yet, in our study, the age- and sex-matched conspecific is only novel in the first conditioning session, then stays the same in all four sessions of social conditioning. Hence, future work should address this limitation in examining the correlations between the estrous cycle of female mice and the social preference to a familiar age- and sex-matched conspecific. Another limitation of our study is that neurotransmitters, mainly serotonin and dopamine (DA), were not measured. Indeed, many studies have implicated DA in same-sex interactions [42,43,44,45]. In particular, it was found that elevating VTA to NAc projection activity and NAc- D1R signaling increases social behavior [44]. Since the serotonin plays a key role in shaping social responses, and the serotonergic system itself is highly responsive to social influences [46], it would be of importance to explore whether differences in the serotonin system could explain the sex differences in social behavior observed in this study. In line with our findings, one study investigating how sex and gonadal hormones modulate sociability and social recognition in C57Bl/6N mice showed that intact male mice investigate conspecifics more than females do, and these differences seem to depend upon circulating hormones released from the testis [47]. Hence, differences in sex hormones might account for the behavioral difference obtained in our study. More research should be conducted to elucidate these possibilities.

In this study, we found that female C57BL6 mice express significantly less social interaction reward than males do. The selected neuropeptides were not sufficient to explain the sex differences in social interaction reward. More research is needed to unravel the candidate(s) underlying this behavioral difference. Our findings have significant translational importance, as several lines of evidence indicate that deficits in social reward are one of the central symptoms and causes of MHD such as depression. Sex differences in the prevalence of this and other MHD might at least in part be due to sex differences in social reward. Therefore, sex differences in social reward and its underlying neural mechanisms are important to address in future research.

## Figures and Tables

**Table 1:** List of primers used to assess relative expression of GOIs using Gapdh as an internal normalization reference.

Primers	Forward (5′–3′)	Reverse (5′–3′)
Gapdh	AGGGCTCATGACCACAGTC	CAGCTCTGGGATGACCTTG
Prepro-orexin	TTCTACAAAGGTTCCCTGGG	CACGTCTTCTGGCGACAG
Oxytocin	ACCTGGATATGCGCAAGTGTCT	ACTGGCAGGGCGAAGGCA
Vasopressin	TCTGCTGCAGCGACGAGA	TTGGCAGAATCCACGGACTC
αCaMKII	AGTCCTACACGAAGATGTGCGA	TCCGGGACCACAGGTTTTCAA

## Data Availability

Data supporting reported results can be found upon request with the corresponding author.
